# Mental health and human rights: never waste a serious crisis

**DOI:** 10.1186/1752-4458-3-12

**Published:** 2009-06-16

**Authors:** Harry Minas

**Affiliations:** 1 Centre for International Mental Health, Melbourne School of Population Health, The University of Melbourne, Parkville, Victoria 3010, Australia

## Abstract

A serious health and human rights crisis is unfolding in Indonesia. Media reports in the Jakarta press have highlighted the high death rates in shelters for people with mental illness that are run by the Jakarta Social Affairs Agency. This crisis represents an opportunity to bring about systematic and substantial changes in the Indonesian mental health system. In order to realise this opportunity the necessary elements of an approach are presented and briefly discussed.

## Never waste a serious crisis

In an interview with the New York Times soon after the US election [1] Barack Obama's Chief of Staff, Rahm Emanuel, said: "Never let a serious crisis go to waste" asserting that crises are "opportunities to do big things." In an interview with the Wall Street Journal [2] he elaborated on the meaning of these statements as he set out a series of essential principles for the Obama administration's program of reform. Emanuel identified previous missed opportunities to deal with major problems, including health, energy, education, fiscal and tax policy, and regulatory reform. "Things we had postponed for too long that were long term are now immediate and must be dealt with ... The problems are big enough that they lend themselves to ideas from both parties for the solution."

## Mentally ill patients dying in Jakarta shelters

During the weekend of 23/24 May 2009 the Jakarta newspapers were focusing on an unfolding crisis in mental health. The headlines in front page and page 2 news stories read: *Mental illness patients die of diarrhea and malnutrition *[3]*Doctors to inspect overcrowded shelters where mentally ill patients are dying *[4]*Mentally ill enter shelters 'for life' *[5]. The flurry of media interest was precipitated by a report from the Jakarta Social Agency that in the city's four 'shelters' for people with chronic mental illness residents are dying of from malnutrition, over-crowding and diarrhoea. "There was not much of an outcry about it; for six months three people every four days died from diarrhea and malnutrition at shelters for mentally ill people in the capital. No fuss was made until the city administration received a report from an agency about the staggering numbers. Recent data from the Jakarta social agency revealed that 181 people died between October 2008 and May 22, 2009, at four shelters in Daan Mogot and Cengkareng, both in West Jakarta, and Cipayung and Ceger, in East Jakarta." [3] A further 57 people who had been transferred from the shelters to the responsible mental hospital had died in the same period. The mortality rate is very high "Out of 644 patients, 53 people died in 2007, 98 died in 2008 and so far 140 people have died this year". [4]

This is not the first time that attention has been brought to the systematic abuse of the human rights of people with mental illness in Indonesia [6-8]. One of the shelters that features in the current news reports was the subject of a story [6] and graphic photoessay [9] in 2003. Abuse of patients in mental hospitals was documented in a report produced by the Department of Psychiatry, University of Indonesia, in 2006 [10]. The restraint and confinement of people with mental illness in the community is receiving overdue attention from researchers [8,11] and governments.

The immediate response to the crisis in the Jakarta shelters is local and piecemeal and does not deal with any of the long-term determinants of the problem. Teams of doctors and paramedics are being dispatched to examine the residents and to provide necessary treatment. "'On Saturday, the team of doctors will go to some of the shelters to check the patients and to bring medicine so there will be no shortage,' said Effendy Anas, the city's assistant to the People's Welfare Office... Dien Emawati, head of Jakarta's Health Agency, said her agency would send on Saturday the team of doctors and officials to the Harapan Sentosa 1 shelter in Cengkareng and the Harapan Sentosa 2 shelter in Cipayung. She said the agency was prioritizing the two shelters but they would consider visiting the smaller shelters. 'The psychiatrists are from Duren Sawit Hospital and the general doctors are from the agency,' Dien said. 'We will also bring sanitary equipment and sanitary officials because it is important for the patients to have a clean environment.' Dien said officials and doctors from local community health centers would also join the team. They would bring medicine to treat mental illness as well as general medicines." [4]

The head of the Jakarta Health Agency has identified over-crowding and insufficient staff as central problems. "Dien said that in addition to the illnesses the patients suffered from, their conditions were worsened by overcrowding in the shelters. She said there were three times as many patients as there were available beds in the facilities. 'It's so crowded, the environment there is bad and has at times led to outbreaks of diarrhea,' she said, adding that her agency had sent essential medicine to the shelters to help treat the problem. Dien said that overcrowding had also forced staff to place aggressive patients in the same room with passive patients, resulting in frequent attacks. She said the malnutrition problem was caused by limited human resources as many mentally ill patients needed to be assisted when eating." [4] A further problem is insufficient funds to feed the shelter residents. The head of Harapan Sentosa 1 shelter in Cengkareng, West Jakarta, "said the current financial budget could not adequately meet patients' needs for better nutritious food. 'We are only given a Rp 15,000 [USD1.5] food budget per patient per day. With the current prices of staple food, it's hard for us to provide nutritious meals."'

## Response to the crisis

The situation of people with mental illness in Indonesia (and elsewhere) is an issue of the kind referred to by Emanuel – "Things we had postponed for too long that were long term are now immediate and must be dealt with" [2]. In relation to the areas requiring major reform in the USA Emanuel makes a vitally important point about collaboration and consensus. "The problems are big enough that they lend themselves to ideas from both parties for the solution" [2].

In any effective response to the problems in the Indonesian mental health system, including the social support arrangements for people with severe and persistent mental illness, several elements are necessary.

1. Civil society groups must unambiguously assert that the life and dignity of a person with mental illness is worth not less than that of any other citizen.

2. The prevalent culture of silence in the face of flagrant and persistent abuse and neglect of the rights of people with mental illness is a major contributor to the current state of affairs and must be changed. Such silence results in a continuing failure to deal with a profound human rights problem and, on moral and legal grounds, cannot be tolerated. Mental health professionals must, wherever possible, prevent such abuses from occurring. When they become aware of such abuses they have a particular responsibility to insist that responsible authorities deal appropriately with them. The recently established Indonesian Mental Health Association (Perhimpunan Jiwa Sehat) can play a vitally imporant role in achieving elements 1 and 2.

3. A broad consensus must be crafted among all stakeholders around two fundamental propositions.

a. The Indonesian government must protect the human rights of people with mental illness, as it is obliged to do under the provisions of the Indonesian constitution [12-14] and the provisions of the international human rights instruments that Indonesia has ratified. Achieving such a consensus will require close collaboration between governments, mental health professional organisations and civil society organisations, particularly consumer and carer associations.

b. The events that have been highlighted by an alert media point to a fundamental problem – the insufficient investment by government in all aspects of the mental health system, including safe and dignified social arrangements for people with mental illness. Solutions to the major problems in the Indonesian mental health system cannot be found without substantial new investment in mental health. Such investment is essential for the provision of acceptable mental health services and social support arrangements.

4. The nature and scope of the human rights abuses being experienced by people with mental illness, and the factors that contribute to the occurrence of such abuses, must be investigated. The National Human Rights Commission should acknowledge that this is a major human rights issue in Indonesia and initiate a systematic and in-depth inquiry. Recommendations from such an inquiry will be vitally important in identifying causes and finding sustainable solutions to this systemic human rights problem.

5. As well as putting the problem of abuse of the human rights of people with mental illness on the national agenda for action recommendations for a coherent plan of action must be developed. This is a process in which the National Taskforce on Mental Health System Development in Indonesia is currently engaged. The Taskforce Working Groups are focused on the areas where mental health system reform is most needed: mental health legislation and policy and mental health system financing; workforce development; community mental health system development; and advocacy and human rights.

6. Currently the problems highlighted by the media are only a mental health problem. While they remain so very little of substance will happen and what does happen will be of the order of providing drugs for treatment of diarrhoea. The nature of the problem must be transformed, re-defined, from a mental health problem to a political problem. An important characteristic of political problems (as opposed to mental health or human rights problems) is that there is usually no problem in finding the money that is necessary to solve them. This is the most important task for mental health advocates, to transform what is now a mental health problem into a political problem.

## Conclusion

What is happening in Indonesia is not unique to that country. It is, if anything, a characteristic and normal state of affairs in many low and middle-income countries across the world [15]. It is of course obvious that the circumstances identified in the Jakarta shelters are a particularly striking and lethal expression of a much deeper malaise and neglect in the Indonesian mental health system. The National Taskforce on Mental Health System Development in Indonesia is well placed to lead the national response to the emerging crisis that has been identified in the recent media reports. This is a serious crisis that should not be wasted.
